# Supplementary material for: Dynamic patterns of functional connectivity in the human brain underlie individual memory formation
Source: Nat Commun. 2024 Oct 17;15:8969. doi: 10.1038/s41467-024-52744-1 (PMC11487248; doi:10.1038/s41467-024-52744-1)
Supplement: Supplementary file 1 — Supplementary Info [file 41467_2024_52744_MOESM1_ESM.pdf]

Supplementary Information

Supplementary Figure 1

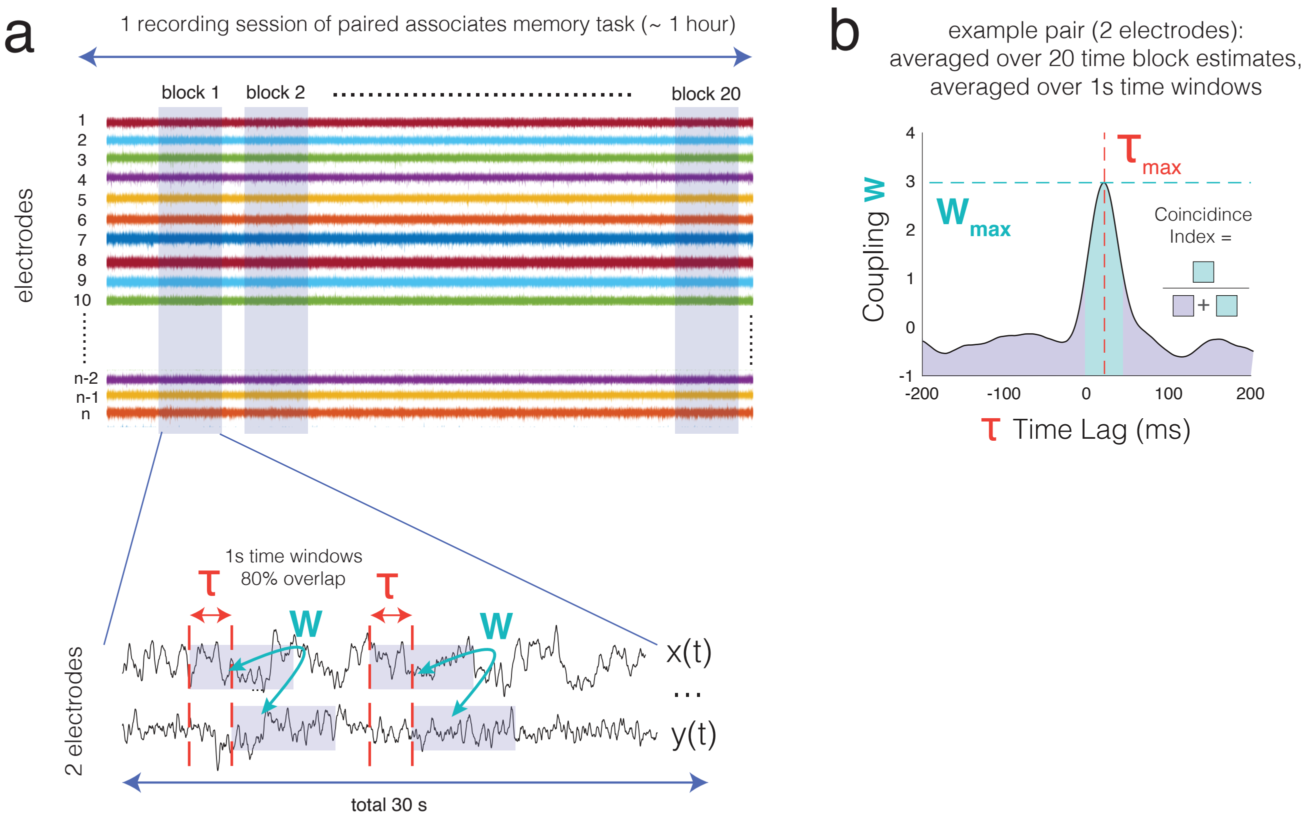

Supplementary Figure 1. Getting the cross-correlation function for an example pair of electrodes across long recording periods. a) We sample 20 time blocks of 30-second data from each recording session. We use a sliding-windows cross-correlation approach to identify electrode pairs exhibiting significant functional connectivity. Next, for every pair of electrodes, we calculate the cross-correlation (coupling strength, W) within 1s windows for all time delays, τ, from -200 to 200 ms. **b)** This generates a cross-correlation function for each electrode pair, averaged over all time blocks and time windows. The cross-correlogram for each electrode pair is characterized by a maximum coupling strength, W_{max}, at a preferred time delay, τ_{max}, and a coincidence index. We select functionally connected electrode pairs based on the distribution of maximum coupling strengths and coincidence indices (see Figure 1E). Source data are provided as a Source Data file.

Supplementary Figure 2

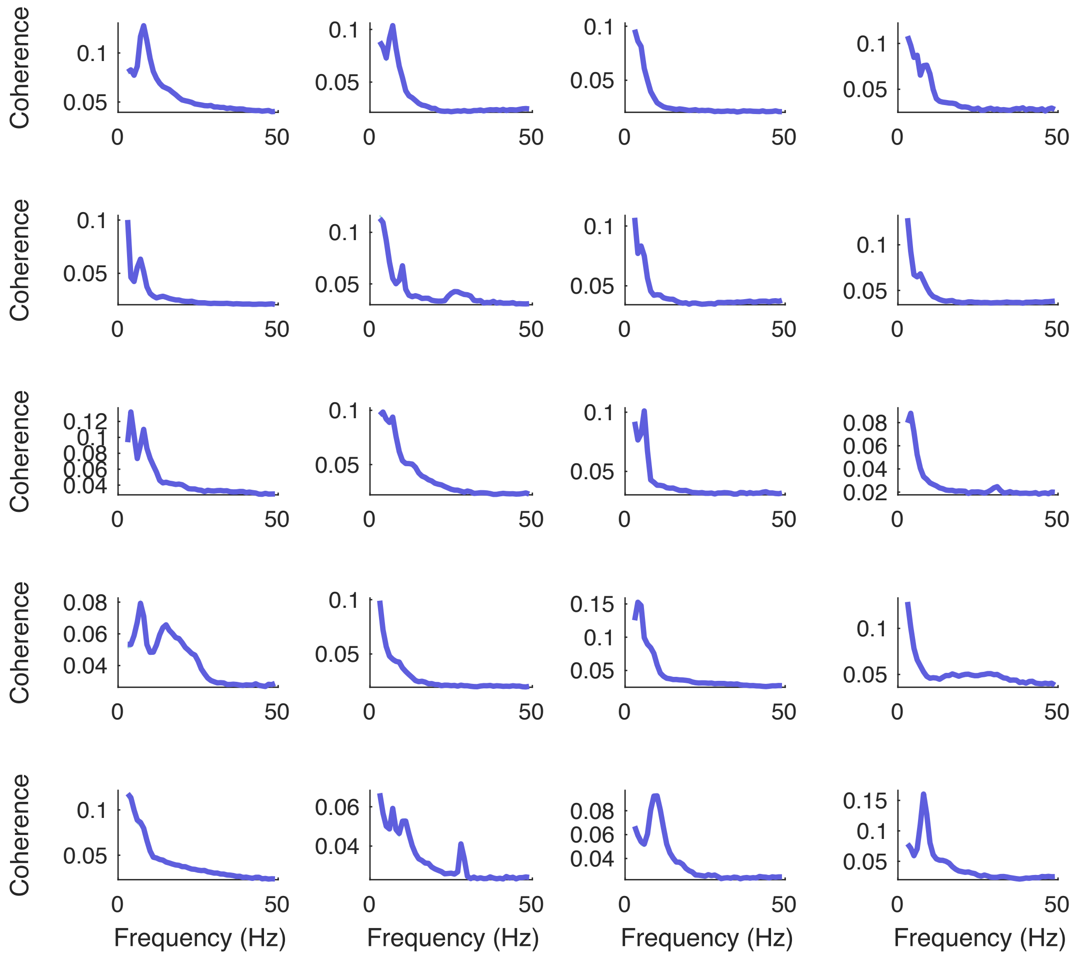

Supplementary Figure 2. Spectral coherence for each participant's functionally connected electrode pairs In each participant (n = 20), we plot the spectral coherence plot averaged across all functionally connected electrode pairs and across randomly selected time blocks throughout recording sessions. Source data are provided as a Source Data file.

Supplementary Figure 3

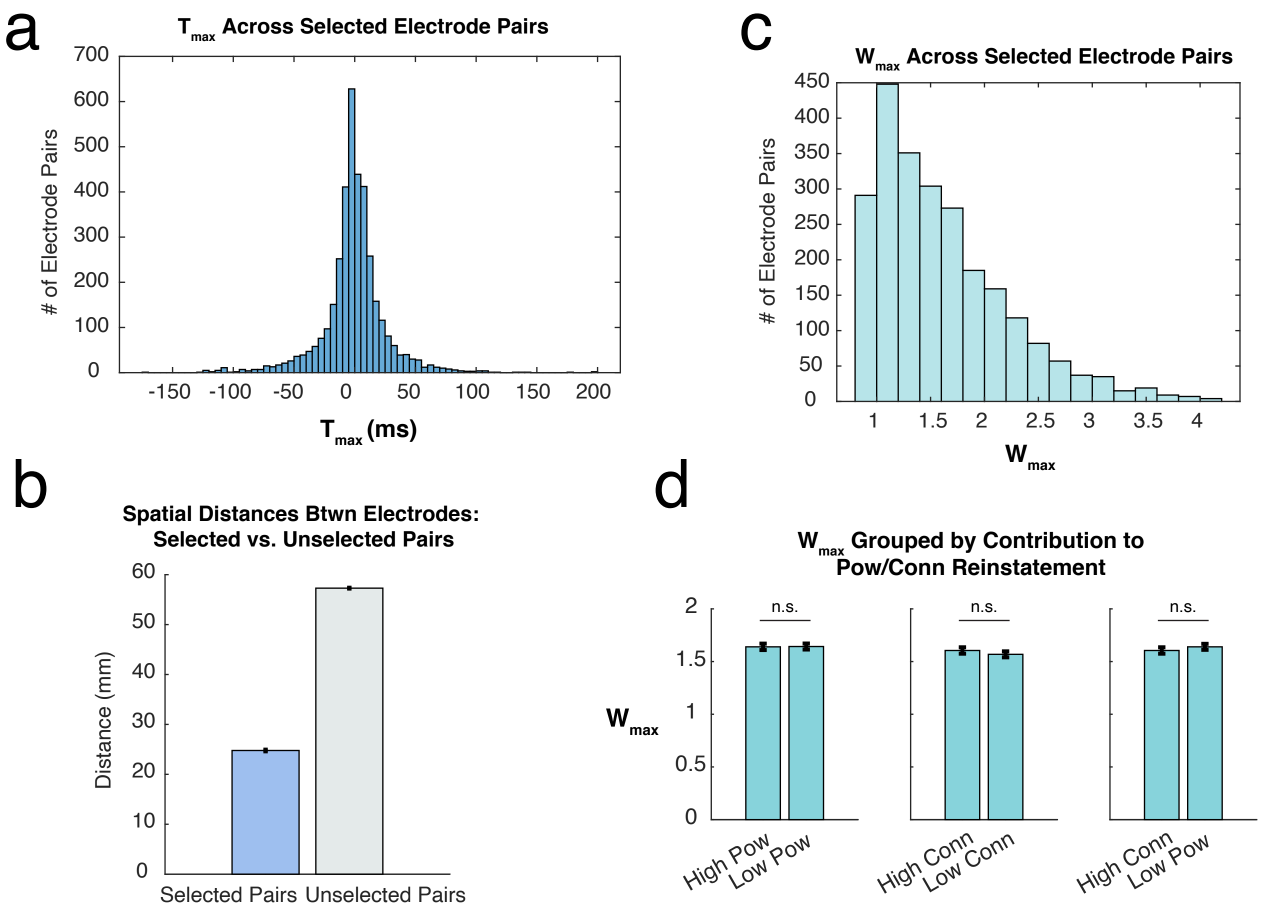

Supplementary Figure 3. Other electrode pair metrics. a) Distribution of τ_{max} across selected electrode pairs. b) Spatial distances (mm) between selected electrode pairs and unselected electrode pairs. Unselected pairs are significantly further apart than selected pairs ($t(127953) = -72.35$, $p < 0.001$). c) Distribution of W_{max} across selected electrode pairs. d) We averaged W_{max} across electrode pairs in which each of the two electrodes fell into the categories of high contribution to power reinstatement, low contribution to power reinstatement, high contribution to connectivity reinstatement, and low contribution to connectivity reinstatement. We compared average W_{max} values among high vs. low contribution to power reinstatement electrode pairs, high vs. low contribution to connectivity reinstatement electrode pairs, and high contribution to connectivity vs. high contribution to power reinstatement electrode pairs, shown below. We did not find a significant difference in average W_{max} values between these groups ($t(817) = -0.07$, $p = 0.94$; $t(840) = 0.93$, $p = 0.35$; $t(854) = -0.86$, $p = 0.39$), suggesting that the W_{max} values of electrode pairs are independent of how they contribute to reinstatement of coupling and spectral power. Source data are provided as a Source Data file.

Supplementary Figure 4

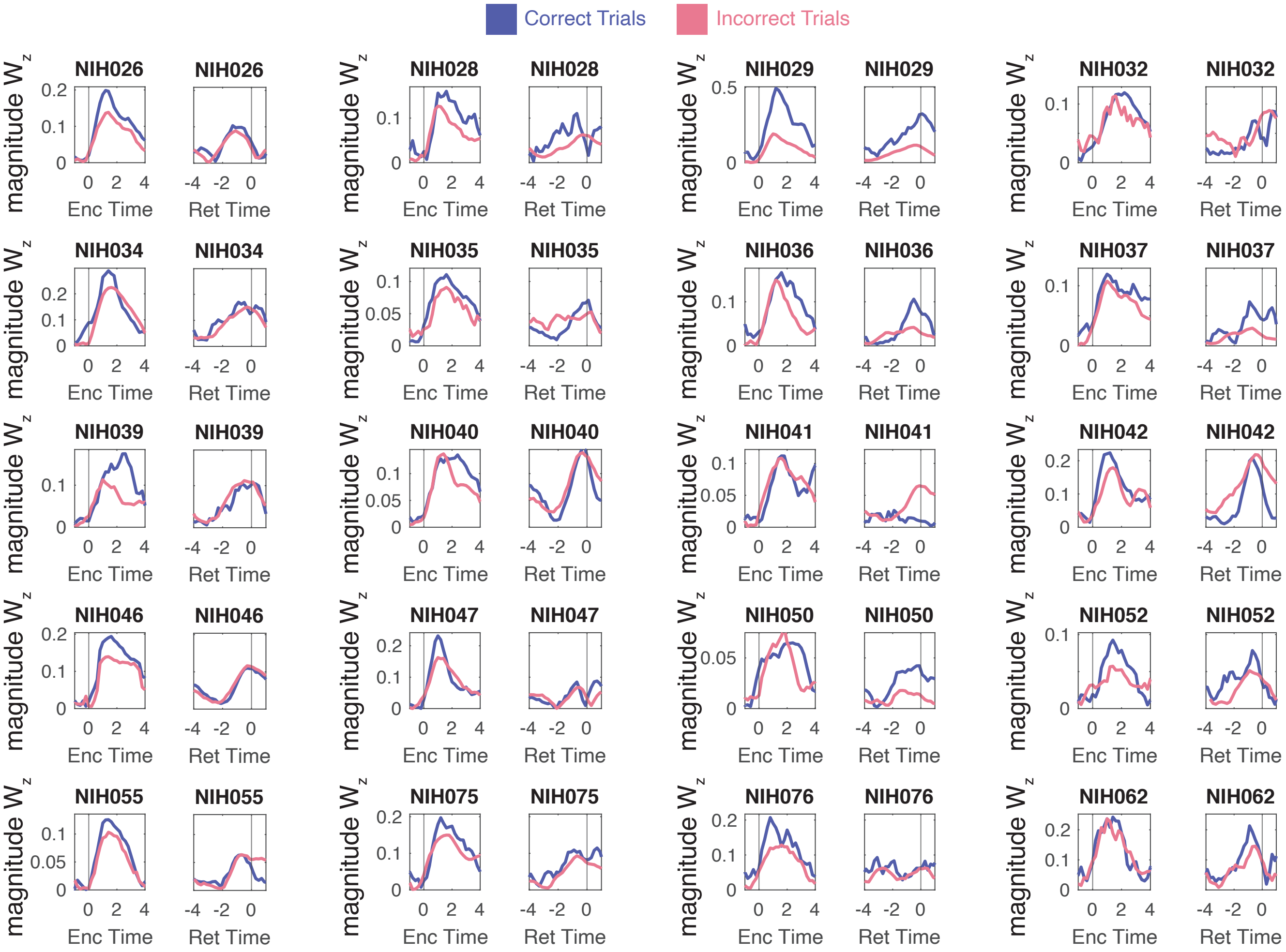

Supplementary Figure 4. Changes in coupling during encoding and retrieval for correct vs. incorrect trials. Time series of changes in the magnitude of coupling, W, for all 20 participants averaged across encoding and retrieval trials, separated by correct trials (blue) and incorrect trials (pink), and averaged across all electrode pairs in each participant. Source data are provided as a Source Data file.

Supplementary Figure 5

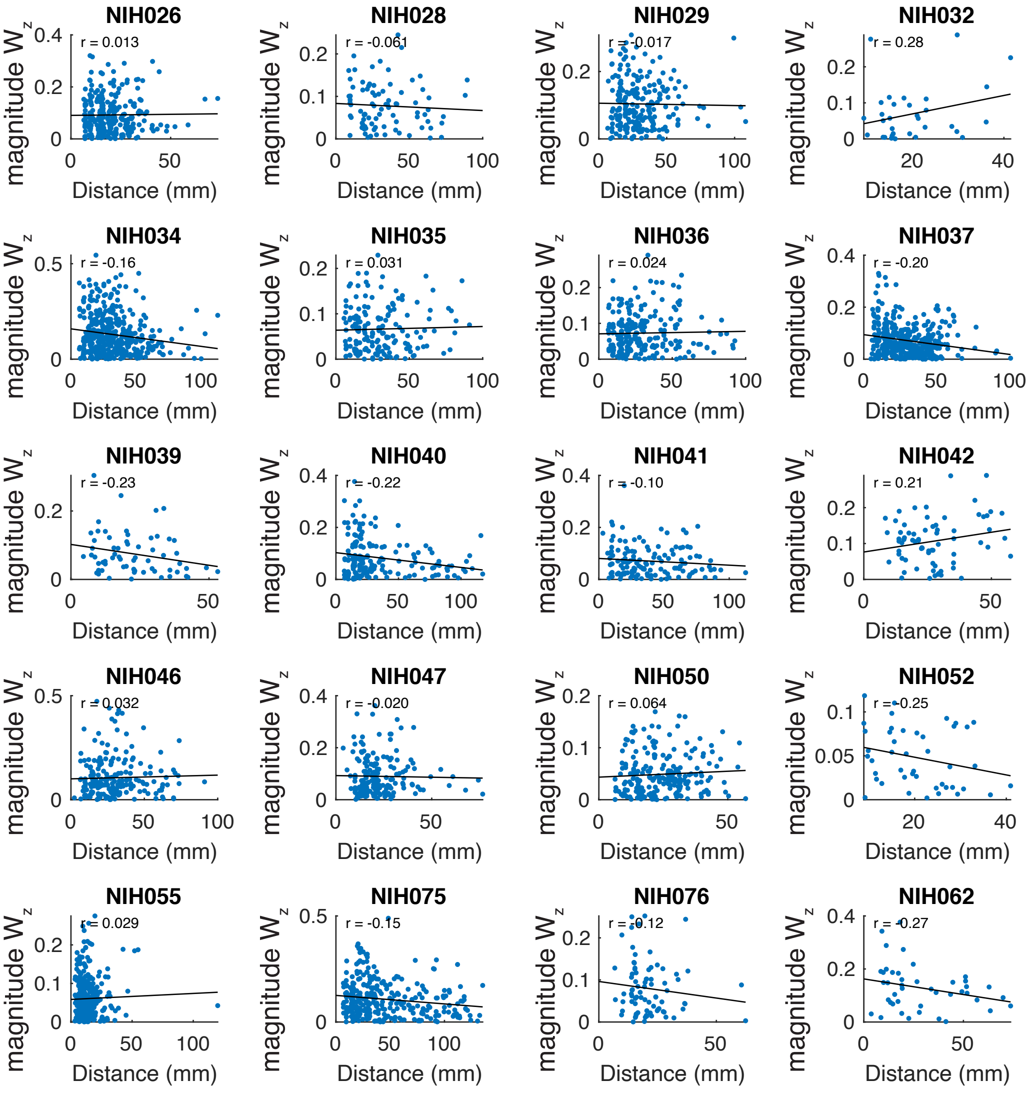

Supplementary Figure 5. Distance between electrodes vs. magnitude of change in coupling. To investigate the relationship between the magnitude of change in coupling and the distance between two functionally connected electrodes, we correlated electrode pairs' magnitude of coupling change relative to baseline (absolute value of z-scored W) during the encoding period after onset and the physical distance (in mm) between two electrodes of an electrode pair. Across participants (n = 20), we did not find a systematic correlation pattern between the magnitude of coupling change and electrode pair distance (average Fisher's transformed $r = -0.057$, $t(19) = 1.69$, $p = 0.11$). Source data are provided as a Source Data file.

Supplementary Figure 6

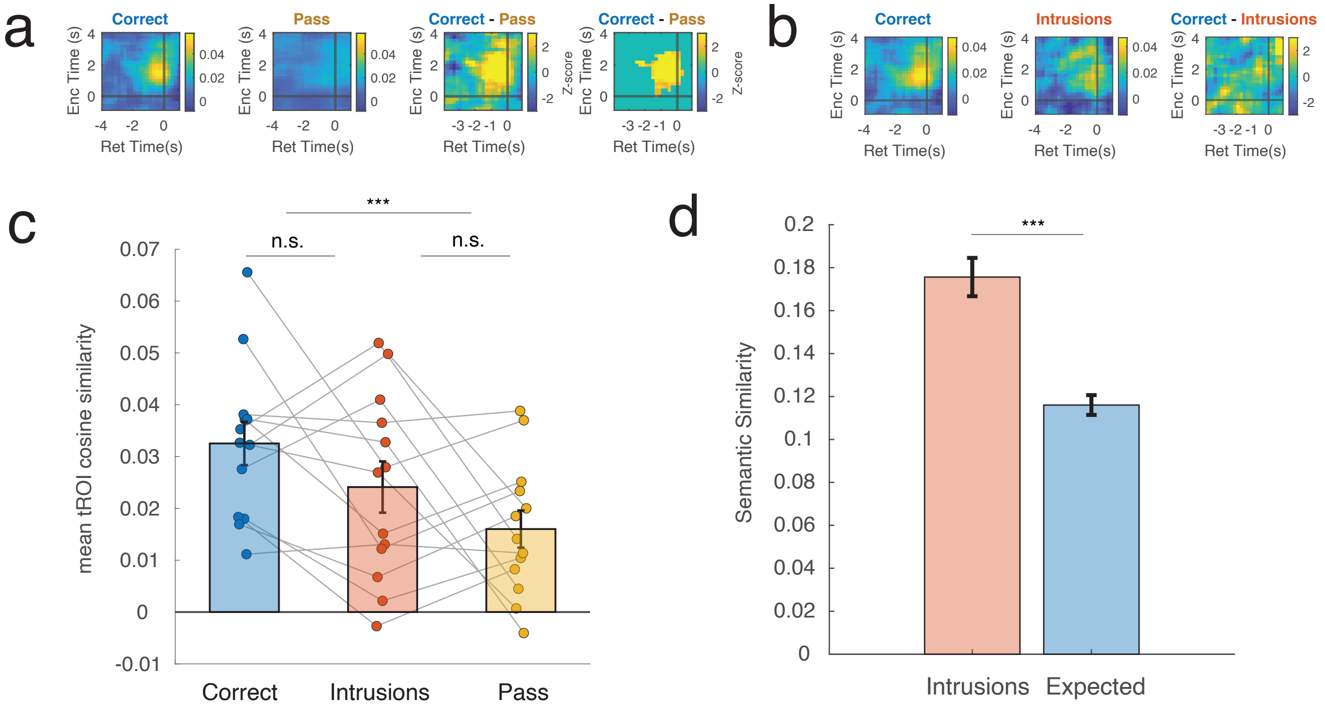

Supplementary Figure 6. Reinstatement effect for pass and intrusion trials. a) Average reinstatement map showing encoding-retrieval similarity across all participants during correct and pass trials, time-locked to study onset for encoding and vocalization for retrieval (cluster-based $p_{corrected} < 0.05$). b) Average reinstatement map showing encoding-retrieval similarity during correct and intrusion trials, time-locked to study onset for encoding and vocalization for retrieval (cluster-based $p_{corrected} < 0.05$). Only participants with at least 10 intrusions trials were included, n = 13. The low intrusion trial counts among participants may contribute to the non-significant result shown (see Supplementary Table S3 for details about number of intrusion trials per participant). c) Mean similarity of patterns of dynamic coupling between encoding and retrieval in the tROI across participants is significantly greater for the correct trials compared to pass trials ($t(12) = 4.31$; $p = 0.0010$), and not significantly greater for the correct trials compared with intrusion trials ($t(12) = 1.62$; $p = 0.13$). Individual participant data shown as dots. Error bars represent SEM across participants. d) Semantic similarity between cue and intruded words is significantly greater than that between cue and expected words ($t(805) = 5.94$; $p = 0.0000000043$). The similarity between neural signals during correct trials and intrusions may be partially attributed to the nature of the intrusions themselves. When people make an intrusion, the intruded word is more semantically similar to the cue word than the expected word. Thus, it could be that when people make an intrusion, they are activating many of the same connectivity patterns that are active when studying the original word pair. Source data are provided as a Source Data file.

Supplementary Figure 7

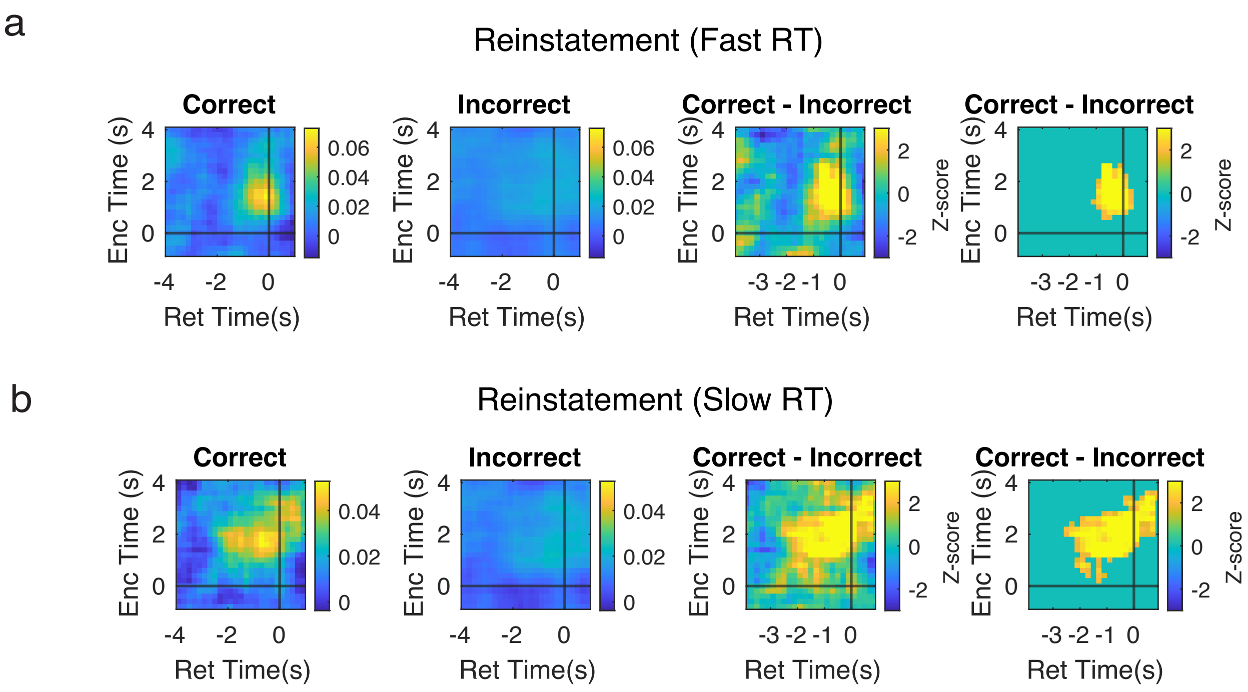

Supplementary Figure 7. Reinstatement analysis for correct trials with fast versus slow reaction times. We analyzed the reinstatement of coupling across all participants (n = 20), splitting each participant's correct trials into fast **(a)** vs. slow **(b)** reaction time trials. For each participant, using that participants median reaction time, we split their correct responses into fast responses (RT less than median RT) or slow responses (RT greater than median RT). Averaging across participants, we see here that the reinstatement effects for fast vs. slow correct responses appear similarly, suggesting that the speed of RT should not affect our results. Source data are provided as a Source Data file.

Supplementary Figure 8

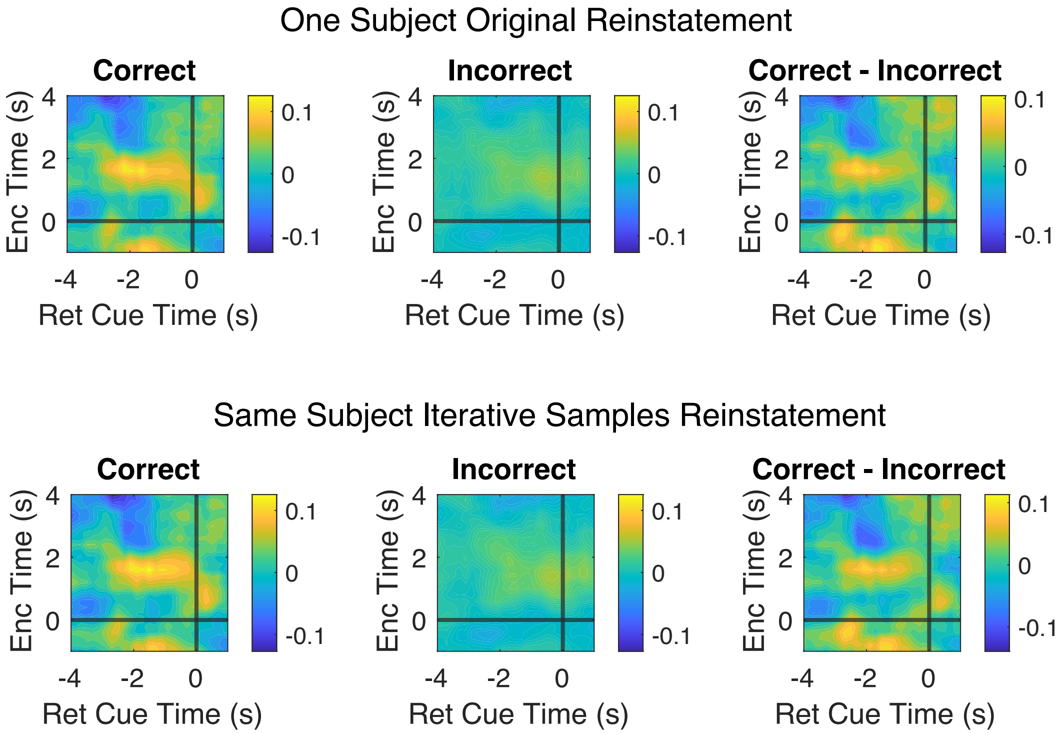

Supplementary Figure 8. Reinstatement analysis for unbalanced correct and incorrect trial counts. We analyzed reinstatement of coupling for one participant (n = 1), the participant with the lowest number of correct trials (n = 13 trials shown on top), and compared it with reinstatement after doing an iterative sampling procedure to account for the unbalanced counts of correct and incorrect trials (shown on bottom). We took samples of n = 13 trials from this participant's correct and incorrect trials and computed reinstatement over 5,000 iterations. We then took the average neural reinstatement across these iterations. We found that the reinstatement profiles appear similarly, suggesting that unbalanced correct and incorrect trial counts should not affect our results. Source data are provided as a Source Data file. Source data are provided as a Source Data file.

Supplementary Figure 9

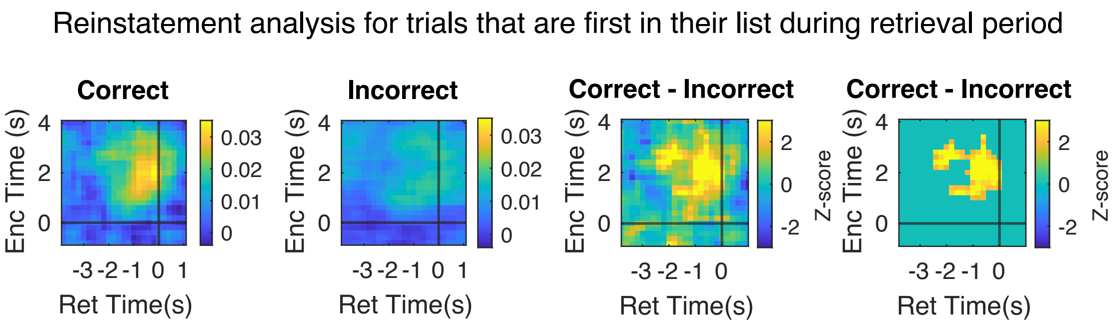

Supplementary Figure 9. Reinstatement analysis for trials that are first in their list during retrieval period. We conducted an analysis to test whether the reinstatement effect persists for only first-presented items in each list at retrieval during the task. We only included participants with at least 10 correct and 10 incorrect first-in-list trials, which was n = 12 participants. Here we show reinstatement maps averaged over these 12 participants. Across these participants, there is significantly greater reinstatement of these patterns of dynamic coupling in correct first-in-list trials as compared with incorrect first-in-list trials, (cluster-based $p_{corrected} < 0.05$; temporal region of interest, tROI; Figure 3c; Supplementary Figure S8). Source data are provided as a Source Data file.

Supplementary Figure 10

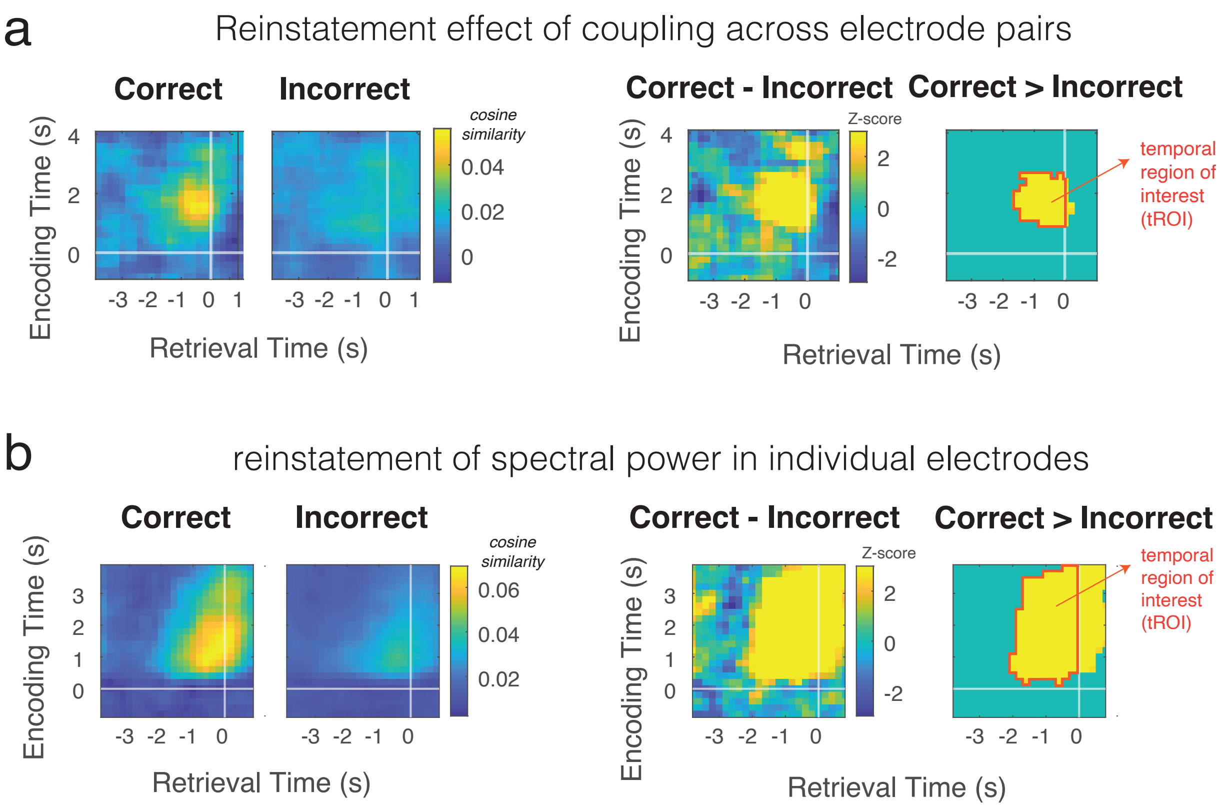

Supplementary Figure 10. Reinstatement effects for coupling and spectral power. Results for the reinstatement effect of coupling in electrode pairs (**A**) and the reinstatement effect of spectral power in individual electrodes (**B**) (n = 20 participants). Shown from left to right for both (A) and (B) is the mean reinstatement across all participants for correct trials, incorrect trials, and their difference (correct incorrect). The highlighted yellow temporal region to the far right denotes encoding-retrieval time pairs during which encoding-retrieval similarity is significantly higher for correct trials compared to incorrect trials. The outlined region in red denotes the tROI's (temporal regions of interest) for both the reinstatement of coupling and the reinstatement of spectral power. We use these two tROI's for our analysis separating the reinstatement of spectral power from the reinstatement of coupling (see Figure 4). Source data are provided as a Source Data file.

Supplementary Figure 11

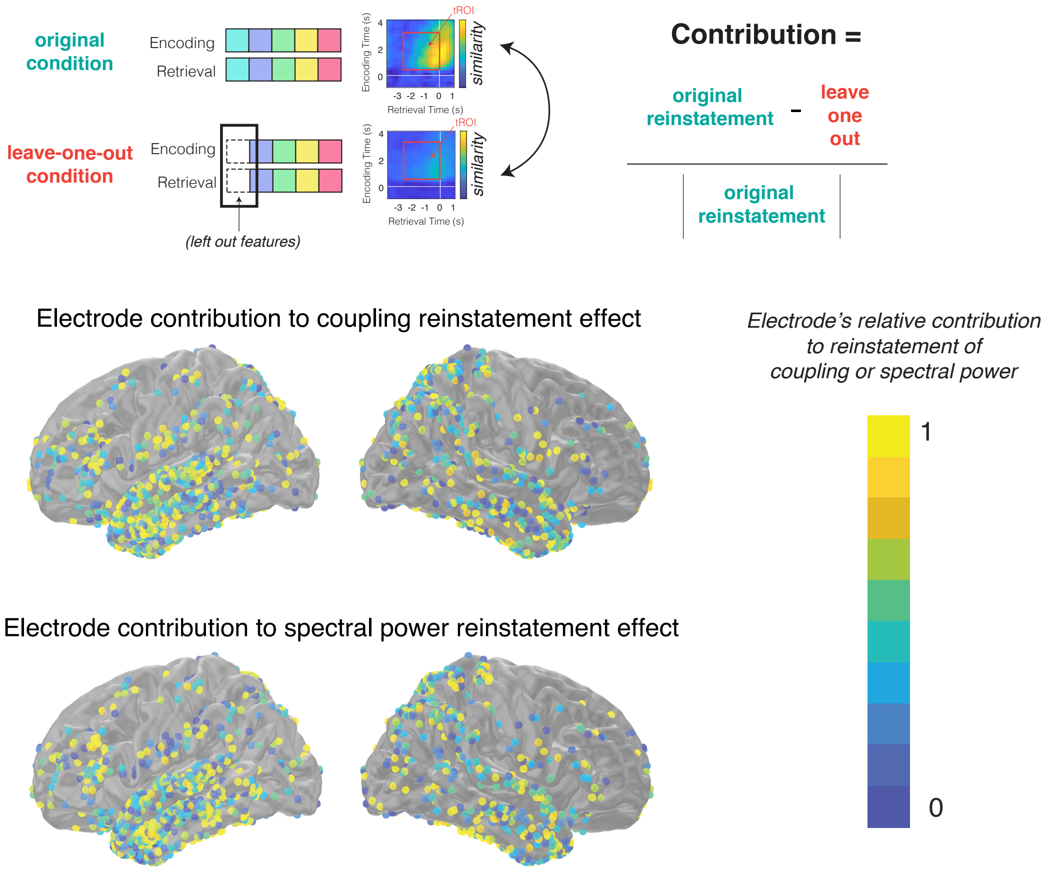

Supplementary Figure 11. Electrode contributions to coupling and spectral power reinstatement. Spatial distribution of electrodes across participants (n = 20) and their relative contributions (percentile) to the reinstatement of spectral power and to the reinstatement of dynamic connectivity. We do not observe any regional localization of brain areas that are more or less important for the reinstatement of spectral power or for the reinstatement of dynamic connectivity. Reinstatement contribution percentiles were calculated using a leave-one-out approach (see Methods). Source data are provided as a Source Data file.

Supplementary Figure 12

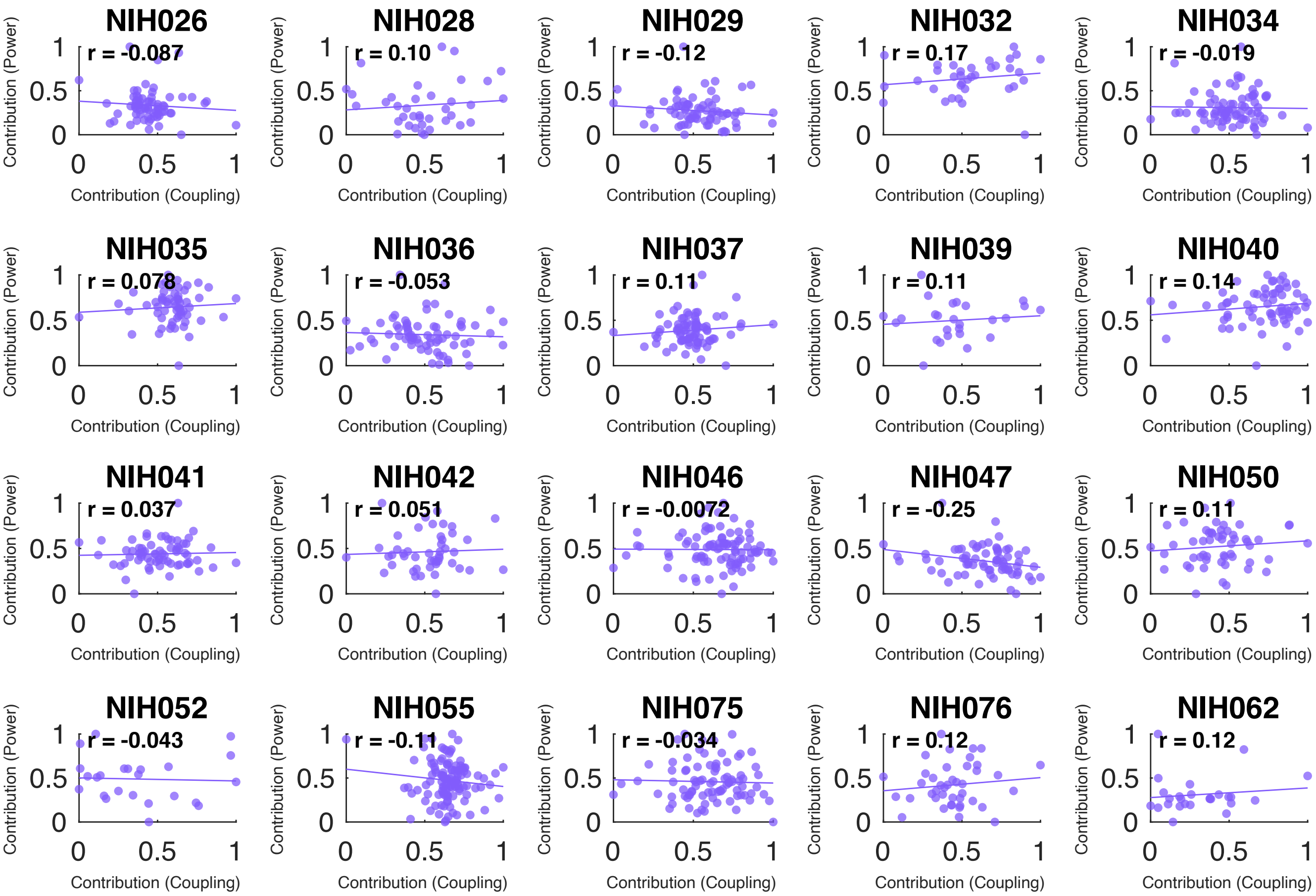

Supplementary Figure 12. Comparing contributions towards connectivity reinstatement vs. power reinstatement. In each participant (n = 20 total participants), we plot every electrode based on its normalized contribution to the reinstatement of coupling and its normalized contribution to the reinstatement of spectral power. We compute a correlation coefficient between these contributions across electrodes. The line of best fit is shown along with r, the participant's correlation coefficient. Across participants, we did not find a systematic correlation pattern (average Fisher's transformed $r = 0.021$, $p = 0.42$, $t(19) = 0.83$). Even after correcting for the attenuation in correlation, this correlation measure remains statistically not significant (average Fisher's transformed $r = 0.051$, $t(19) = 0.67$, $p = 0.51$). Source data are provided as a Source Data file.

Supplementary Figure 13

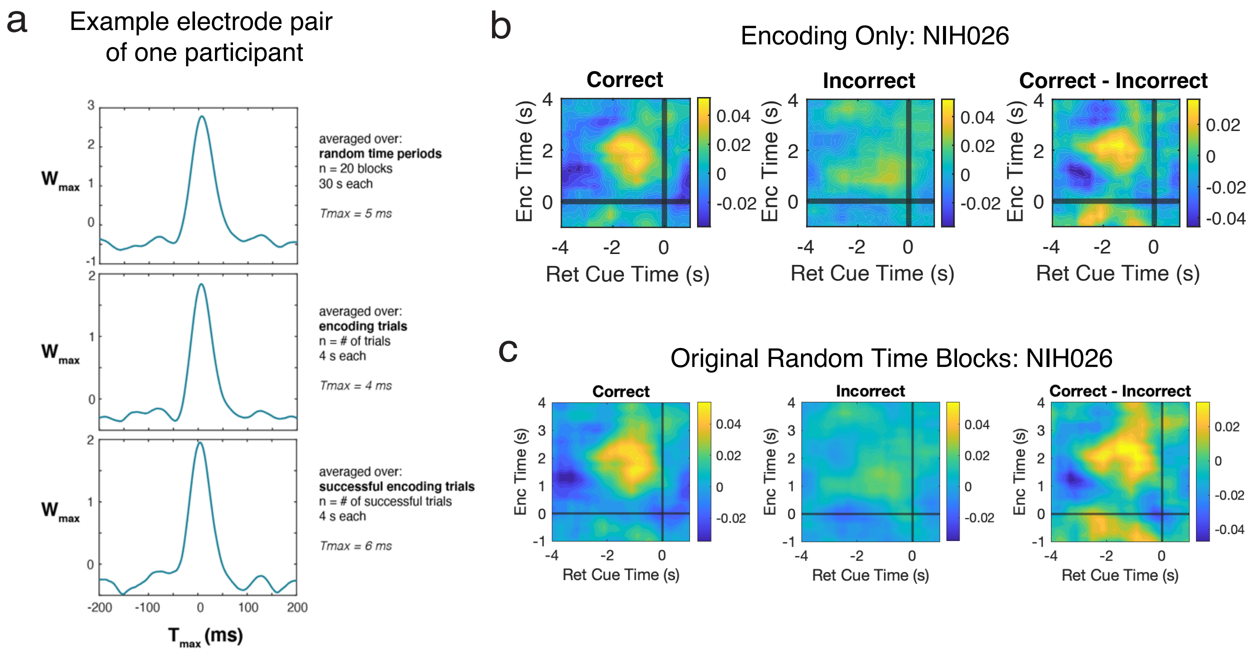

Supplementary Figure 13. Sampling from task time periods versus random time periods. a) We show below the coupling profile for one example electrode pair of one participant (n = 1 participant), generated using three different time sampling methods: first, our original method shown in the paper taking time blocks across the recording session; second, taking time blocks only from encoding trials; and third, taking time blocks only during successful encoding trials. Across the three different methods, there is a very reliable and stable coupling profile for this pair, suggesting that the profiles we have identified are consistent whether we use time blocks distributed throughout the entire task or only times during the encoding trials. **b)** We also computed reinstatement analysis of coupling for one participant when sampling time blocks only from encoding trials. **c)** Our original reinstatement analysis using the original sampling from random time periods for this participant. These results suggest that these results exhibit the same relative insensitivity to sampling from trial time periods. Source data are provided as a Source Data file.

739 **Supplementary Figure 14**

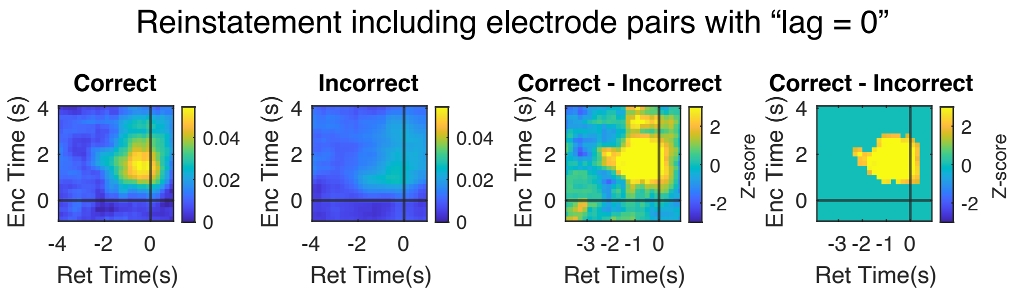

Supplementary Figure 14. Reinstatement of coupling after including electrode pairs with lag = 0 (less than |1| ms). Some electrode pairs we excluded in our original analysis with lag less than |1| ms may be true connections and not due to volume conduction. To test this, we recomputed reinstatement of coupling for across all participants (n = 20), including electrode pairs with lag = 0. These results show that our main reinstatement effect appears similar with or without lag = 0 connections. Source data are provided as a Source Data file.

Table S1. Demographic information and clinical characteristics of the participants in the iEEG sample.

Subject ID	Age (Range)	Sex	IQ	Language Dominance	Resection
NIH026	20-34	F	80	L	L temporal lobectomy w/ amygdalohippocampectomy
NIH028	20-34	M	106	L	L temporal lobectomy, amygdalohippocampectomy
NIH029	20-34	M	75	L	None
NIH032	20-34	F	94	L	R temporal lobectomy, amygdalohippocampectomy
NIH034	45-54	M	74	L	L selective amygdalohippocampectomy
NIH035	35-44	F	79	L	R inferior, middle temporal gyrus corticectomy
NIH036	20-34	M	91	L	L temporal lobectomy w/ amygdalohippocampectomy, Resection of periventricular heterotopia
NIH037	35-44	F	92	L	L temporal lobectomy w/ amygdalohippocampectomy
NIH039	20-34	F	86	L	L temporal lobectomy w/ amygdalohippocampectomy
NIH040	20-34	F	79	L	R temporal lobectomy w/ amygdalohippocampectomy
NIH041	20-34	M	74	L	R parietal corticectomy
NIH042	20-34	M	78	L	L superior temporal corticectomy
NIH046	20-34	M	N/A	L	R parietal topectomy
NIH047	20-34	M	88	L & R	L temporal lobectomy, amygdalohippocampectomy, resection of heteropia
NIH050	20-34	M	78	L	R temporal lobectomy, amygdalohippocampectomy
NIH052	55-64	M	108	L	R temporal lobectomy, amygdalohippocampectomy
NIH055	20-34	F	76	L	R parietal topectomy
NIH062	20-34	M	106	L	None
NIH075	20-34	M	87	L	L anterior temporal lobectomy and amygdalohippocampectomy
NIH076	45-54	F	N/A	L	L anterior temporal lobectomy and orbitofrontal topectomy
**Mean +/- SEM**	**33.40 +/- 2.26**	N/A	**86.17 +/- 2.67**	N/A	N/A

Note. R = Right; L = Left. a). All participants' data were used for the behavioral analysis. b) Participants should have at least 3 electrodes after artifacts correction with a minimum of 10 accurate recall trials to be included for the iEEG analysis.

Table S2. Behavioral memory task results and electrode details for each participant.

Subject ID	Total Trials	Correct Trials	Incorrect Trials	Intrusion Trials	Pass Trials	Median Reaction Time (ms)	Electrodes (n)	All Possible Electrode Pairs (n(n-1)/2)	Functionally Connected Electrode Pairs	% of Functionally Connected Pairs Out of All Possible Pairs
NIH026	291	119	172	14	158	2031	82	3321	271	8.16
NIH028	296	39	257	2	255	2204	44	946	85	8.99
NIH029	297	19	278	7	271	2430	81	3240	218	6.73
NIH032	300	242	58	9	49	945	77	2926	32	1.09
NIH034	135	13	122	32	90	2662	87	3741	407	10.88
NIH035	287	190	97	16	81	1818	72	2556	172	6.73
NIH036	271	35	236	31	205	2869	80	3160	238	7.53
NIH037	298	22	276	2	274	2323	84	3486	397	11.39
NIH039	296	59	237	47	190	2343	61	1830	63	3.44
NIH040	298	106	192	36	156	1566	77	2926	170	5.81
NIH041	294	40	254	49	205	1773	76	2850	134	4.70
NIH042	269	121	148	23	125	1672	108	5778	70	1.21
NIH046	291	140	151	21	130	1840	104	5356	221	4.13
NIH047	296	75	221	45	176	1527	88	3828	186	4.86
NIH050	287	198	89	4	85	2162	70	2415	216	8.94
NIH052	277	84	193	10	183	2485	31	465	44	9.46
NIH055	198	87	111	18	93	2310	132	8646	334	3.86
NIH062	116	17	99	19	80	2780	87	3741	311	8.31
NIH075	135	21	114	10	104	2147	53	1378	80	5.81
NIH076	65	17	48	9	39	2173	42	861	43	4.99
**Mean +/- SEM**	**249.85 +/- 16.75**	**82.20 +\- 15.26**	**167.65 +/- 16.54**	**20.20 +/- 3.36**	**147.45 +/- 71.39**	**2103 +/- 105$**	**76.80 +/- 5.25**	**3172.50 +/- 418.49**	**184.60 +/- 26.23**	**6.35% +/- 0.65%**

Table S3. Session and electrode pair details for each participant.

Participants	Session Number	Time Between Sessions	All Possible Pairs	Selected Pairs	% Selected Pairs
NIH026	NIH026 session 1	23 h, 6 min	3655	491	13.43
	NIH026 session 2		3486	409	11.73
NIH028	NIH028 session 1	4 h, 58 min	946	118	12.47
	NIH028 session 2		946	137	14.48
NIH029	NIH029 session 1	1 day, 1 h, 59 min	3486	486	13.94
	NIH029 session 2		4095	509	12.43
NIH032	NIH032 session 1	47 h, 6 min	3486	173	4.96
	NIH032 session 2		3916	193	4.93
NIH034	NIH034 session 1	N/A	3741	465	12.43
NIH035	NIH035 session 1	3 h, 58 min	2556	265	10.37
	NIH035 session 2		2628	288	10.96
NIH036	NIH036 session 1	3 h, 13 min	3160	356	11.27
	NIH036 session 2		3160	390	12.34
NIH037	NIH037 session 1	5 h, 26 min	3741	593	15.85
	NIH037 session 2		3486	495	14.2
NIH039	NIH039 session 1	1 day, 3 h, 45 min	1830	262	14.32
	NIH039 session 2		2346	349	14.88
NIH040	NIH040 session 1	3 h, 1 min	2926	290	9.91
	NIH040 session 2		2926	286	9.77
NIH041	NIH041 session 1	1 day, 2 h, 47 min	2850	304	10.67
	NIH041 session 2		3403	401	11.78
NIH042	NIH042 session 1	4 days, 4 h, 10 min	6670	841	12.61
	NIH042 session 2		7021	436	6.21
NIH046	NIH046 session 1	6 h, 27 min	5671	487	8.59
	NIH046 session 2		5778	427	7.39
NIH047	NIH047 session 1	3 h, 25 min	4095	379	9.26
	NIH047 session 2		4005	314	7.84
NIH050	NIH050 session 1	18 days, 23 h, 39 min	2485	631	25.39
	NIH050 session 2		2628	390	14.84
NIH052	NIH052 session 1	14 h, 33 min	465	66	14.19
	NIH052 session 2		528	80	15.15
NIH055	NIH055 session 1	48 h, 3 min	9180	946	10.31
	NIH055 session 2		9453	774	8.19
NIH062	NIH062 session 1	N/A	435	43	9.89
NIH075	NIH075 session 1	N/A	3741	311	8.31
NIH076	NIH076 session 1	2 days, 3 h, 58 min	1653	137	8.29
	NIH076 session 2		1431	223	15.58
**Mean +/- SEM**	**Mean +/- SEM**	**24.15 hours +/- 5.5 hours**	**3459.7 +/- 344.83**	**371.49 +/- 33.93**	**11.60 +/- 0.62**

Table S4. Anatomical information of functionally connected electrode pairs

Electrode Region 1	Electrode Region 2	# Identified Pairs	# Possible Pairs	Percent % of Identified/Possible Pairs
Posterior/Occipital	Posterior/Occipital	854	10006	8.5349
Posterior/Occipital	Posterior Temporal Lobe	190	3971	4.7847
Posterior/Occipital	MTL	30	1348	2.2255
Posterior/Occipital	Anterior Temporal Lobe	170	5693	2.9861
Posterior/Occipital	Frontal	36	1039	3.4649
Posterior Temporal Lobe	Posterior Temporal Lobe	145	1102	13.1579
Posterior Temporal Lobe	MTL	53	753	7.0385
Posterior Temporal Lobe	Anterior Temporal Lobe	485	4573	10.6057
Posterior Temporal Lobe	Frontal	30	793	3.7831
MTL	MTL	88	323	27.2446
MTL	Anterior Temporal Lobe	199	1923	10.3484
MTL	Frontal	10	287	3.4843
Anterior Temporal Lobe	Anterior Temporal Lobe	1209	7707	15.687
Anterior Temporal Lobe	Frontal	124	1203	10.3076
Frontal	Frontal	69	505	13.6634
