# Supplementary material for: Dynamic patterns of functional connectivity in the human brain underlie individual memory formation
Source: Nat Commun. 2024 Oct 17;15:8969. doi: 10.1038/s41467-024-52744-1 (PMC11487248; doi:10.1038/s41467-024-52744-1)
Supplement: Supplementary file 3 — Reporting Summary [file 41467_2024_52744_MOESM3_ESM.pdf]

nature portfolio

Corresponding author(s): Kareem A. Zaghloul

Last updated by author(s): August 25, 2024

Reporting Summary

Nature Portfolio wishes to improve the reproducibility of the work that we publish. This form provides structure for consistency and transparency in reporting. For further information on Nature Portfolio policies, see our Editorial Policies and the Editorial Policy Checklist.

Statistics

For all statistical analyses, confirm that the following items are present in the figure legend, table legend, main text, or Methods section.

n/a	Confirmed	
☐	☒	The exact sample size (n) for each experimental group/condition, given as a discrete number and unit of measurement
☐	☒	A statement on whether measurements were taken from distinct samples or whether the same sample was measured repeatedly
☐	☒	The statistical test(s) used AND whether they are one- or two-sided<br>*Only common tests should be described solely by name; describe more complex techniques in the Methods section.*
☐	☒	A description of all covariates tested
☐	☒	A description of any assumptions or corrections, such as tests of normality and adjustment for multiple comparisons
☐	☒	A full description of the statistical parameters including central tendency (e.g. means) or other basic estimates (e.g. regression coefficient) AND variation (e.g. standard deviation) or associated estimates of uncertainty (e.g. confidence intervals)
☐	☒	For null hypothesis testing, the test statistic (e.g. F, t, r) with confidence intervals, effect sizes, degrees of freedom and P value noted<br>*Give P values as exact values whenever suitable.*
☒	☐	For Bayesian analysis, information on the choice of priors and Markov chain Monte Carlo settings
☒	☐	For hierarchical and complex designs, identification of the appropriate level for tests and full reporting of outcomes
☐	☒	Estimates of effect sizes (e.g. Cohen's d, Pearson's r), indicating how they were calculated

Our web collection on statistics for biologists contains articles on many of the points above.

Software and code

Policy information about availability of computer code

Data collection	iEEG data were acquired using Nihon Khoden's or Blackrock's EEG data acquisition software. Behavioral data for iEEG participants were collected using Python 2.0 code.
Data analysis	Electrode localization was fulfilled by using AFNI and FreeSurfer 6.0 software packages. All visualization and statistical analyses were performed using custom code in Matlab 2021a (Mathworks, Inc.). Custom code is available for public download at https://research.ninds.nih.gov/zaghloul-lab/downloads.

For manuscripts utilizing custom algorithms or software that are central to the research but not yet described in published literature, software must be made available to editors and reviewers. We strongly encourage code deposition in a community repository (e.g. GitHub). See the Nature Portfolio guidelines for submitting code & software for further information.

Data

Policy information about availability of data

All manuscripts must include a data availability statement. This statement should provide the following information, where applicable:

- Accession codes, unique identifiers, or web links for publicly available datasets
- A description of any restrictions on data availability
- For clinical datasets or third party data, please ensure that the statement adheres to our policy

The data that support the findings of this study are available for public download at https://research.ninds.nih.gov/zaghloul-lab/downloads.

Research involving human participants, their data, or biological material

Policy information about studies with human participants or human data. See also policy information about sex, gender (identity/presentation), and sexual orientation and race, ethnicity and racism.

Reporting on sex and gender	No sex or gender based analyses were performed as they were not relevant to this study's research questions.
Reporting on race, ethnicity, or other socially relevant groupings	No race or ethnicity based analyses were performed as they were not relevant to this study's research questions.
Population characteristics	Twenty participants (8 females; 33.40 ± 2.30 years old; Wechsler Intelligence Quotient [IQ]: 88.17 ± 2.67 , all > 70; see Table S1 for more details) with drug-resistant epilepsy implanted with subcortical electrodes for seizure monitoring.
Recruitment	Patients were recruited to the NIH based on candidacy for protocol (must have drug-resistant epilepsy). Patients were recruited by self-referral, and by referral from healthcare providers in the community and nationwide. Our center provides free treatment irrespective of insurance status, and so we recruit patients across the spectrum of socio-economic status. We do not expect substantial bias stemming from referral patterns or self-selection. Participants were not financially compensated for participating in our behavioral tasks.
Ethics oversight	The Institutional Review Board of the National Institutes of Health and the National Institute of Neurological Disorders and Stroke approved the experimental protocol (11-N-0051 Epilepsy Surgery) through which these data were collected.

Note that full information on the approval of the study protocol must also be provided in the manuscript.

Field-specific reporting

Please select the one below that is the best fit for your research. If you are not sure, read the appropriate sections before making your selection.

- [x] Life sciences
- [ ] Behavioural & social sciences
- [ ] Ecological, evolutionary & environmental sciences

For a reference copy of the document with all sections, see nature.com/documents/nr-reporting-summary-flat.pdf

Life sciences study design

All studies must disclose on these points even when the disclosure is negative.

Sample size	No upfront sample size calculation was performed. Sample size is based on previous studies of human cognitive function using iEEG data. Twenty participants (8 females; 33.40 ± 2.30 years old; Wechsler Intelligence Quotient [IQ]: 88.17 ± 2.67 , all > 70; see Table S1) with drug resistant epilepsy underwent a surgical procedure at the Clinical Center of the National Institutes of Health (NIH, Bethesda, Maryland, USA), and participated in this study.
Data exclusions	We used the following inclusion criteria for the analysis of neural data: 1) participants should have no prior resection of brain regions; 2) they should have completed at least 10 accurate trials of the memory task across experimental sessions; and 3) the recorded intracranial EEG data should not contain excessive environmental noise or motor artifacts.
Replication	Our study is retrospective, so prospective replication of the results is not feasible. However, we do use statistical tests to ensure that trends across patients observed in our data are robust. These are described in our Methods section.
Randomization	Randomization of participants was not relevant to this study and participants were therefore not allocated into separate groups.<br>Every participant completed an experimental session with task stimuli randomly chosen from a word pool of 300 words. All experimental factors, such as study order and test order, were fully randomized within list.
Blinding	Blinding was not relevant to this study as there was no group allocation for our analysis.

Reporting for specific materials, systems and methods

We require information from authors about some types of materials, experimental systems and methods used in many studies. Here, indicate whether each material, system or method listed is relevant to your study. If you are not sure if a list item applies to your research, read the appropriate section before selecting a response.

Materials & experimental systems

n/a	Involved in the study
☒	☐ Antibodies
☒	☐ Eukaryotic cell lines
☒	☐ Palaeontology and archaeology
☒	☐ Animals and other organisms
☒	☐ Clinical data
☒	☐ Dual use research of concern
☒	☐ Plants

Methods

n/a	Involved in the study
☒	☐ ChIP-seq
☒	☐ Flow cytometry
☒	☐ MRI-based neuroimaging
